# A General Framework for Inferring Bayesian Ideal Observer Models from Psychophysical Data

**DOI:** 10.1523/ENEURO.0144-22.2022

**Published:** 2023-01-06

**Authors:** Tyler S. Manning, Benjamin N. Naecker, Iona R. McLean, Bas Rokers, Jonathan W. Pillow, Emily A. Cooper

**Affiliations:** 1 Herbert Wertheim School of Optometry and Vision Science, University of California, Berkeley, Berkeley, CA 94720; 2 Psychology, University of Texas at Austin, Austin, TX 78712; 3 Psychology, New York University–Abu Dhabi, Abu Dhabi, United Arab Emirates; 4 Princeton Neuroscience Institute, Department of Psychology, Princeton University, Princeton, NJ 08540; 5 Herbert Wertheim School of Optometry and Vision Science, Helen Wills Neuroscience Institute, University of California, Berkeley, Berkeley, CA 94720

**Keywords:** ideal observer models, perception, Bayesian inference

## Abstract

A central question in neuroscience is how sensory inputs are transformed into percepts. At this point, it is clear that this process is strongly influenced by prior knowledge of the sensory environment. Bayesian ideal observer models provide a useful link between data and theory that can help researchers evaluate how prior knowledge is represented and integrated with incoming sensory information. However, the statistical prior employed by a Bayesian observer cannot be measured directly, and must instead be inferred from behavioral measurements. Here, we review the general problem of inferring priors from psychophysical data, and the simple solution that follows from assuming a prior that is a Gaussian probability distribution. As our understanding of sensory processing advances, however, there is an increasing need for methods to flexibly recover the shape of Bayesian priors that are not well approximated by elementary functions. To address this issue, we describe a novel approach that applies to arbitrary prior shapes, which we parameterize using mixtures of Gaussian distributions. After incorporating a simple approximation, this method produces an analytical solution for psychophysical quantities that can be numerically optimized to recover the shapes of Bayesian priors. This approach offers advantages in flexibility, while still providing an analytical framework for many scenarios. We provide a MATLAB toolbox implementing key computations described herein.

## Significance Statement

Ideal observer models in neuroscience are an important tool for developing and testing hypotheses about how sensory information is processed. Here, we review the canonical application of Bayesian ideal observer models for understanding sensory processing. We present a new mathematical generalization that will allow these models to be used for deeper investigations into how prior knowledge influences perception. We also provide a software toolkit for implementing the described models.

## Introduction

Sensory systems must encode information about environmental stimuli in a way that supports successful behaviors. However, sensory measurements are often noisy and ambiguous, making this a demanding task. For example, in the visual system, each retinal image is consistent with an infinite number of possible three-dimensional scenes. In the auditory system, the vibration of the inner ear intermixes both the identity and elevation of sound sources. Prior knowledge about the environment can help resolve these ambiguities (Knill and Richards, 1996; Simoncelli and Olshausen, 2001). Thus, advances in understanding sensation and perception often rely on understanding how prior knowledge is represented in the nervous system and how this prior knowledge influences our percepts.

The influence of prior knowledge on perception is often characterized using psychophysical experiments that measure the bias and variability of perceptual reports (Hürlimann et al., 2002; Weiss et al., 2002; Adams et al., 2004; Girshick et al., 2011; Vacher et al., 2018). For example, measured biases can be compared with biases predicted by ideal observer models, which can also inform our understanding of how sensory information is represented within neuronal populations (Ganguli and Simoncelli, 2010; Wei and Stocker, 2015, 2017; Morais and Pillow, 2018). Bayesian ideal observer models specifically posit that observers optimally combine noisy sensory measurements with a probability distribution representing the relative frequency with which events occur in the world (called the prior distribution, or simply the prior). Bayesian models are popular across many domains, including sensation and perception, because they can successfully explain a wide range of phenomena (Weiss et al., 2002; Adams et al., 2004; Burge et al., 2010; Girshick et al., 2011; Kim and Burge, 2018). However, these models are often poorly constrained. Without constraints on the shape of the prior, Bayesian models can effectively explain any biases. Thus, a set of important questions arise: What is the shape of the prior the observer is using? Does this shape accurately reflect probabilities in the world? Does it change systematically with experience?

Bayesian priors are often assumed to take the form of a Gaussian distribution for computational efficiency (Mamassian and Landy, 1998; Weiss et al., 2002; Beierholm et al., 2009; Sotiropoulos et al., 2011; Saunders and Chen, 2015; Rokers et al., 2018). This assumption, however, limits the ability to ask questions about the shape of the prior because a Gaussian only has two parameters. In addition, analyses of natural scene statistics suggest that the probability distributions of environmental stimuli are generally non-Gaussian (Dong and Atick, 1995; Girshick et al., 2011; Sprague et al., 2015). In order to more flexibly model prior distributions, a previous study introduced an analytic approach based on piecewise approximations that leverages assumptions about the local shape of the prior relative to the magnitude of measurement noise (Stocker and Simoncelli, 2006). An alternative approach to increasing flexibility without introducing assumptions about prior shape is to use numeric methods that do not place constraints on the global parametric form or local properties of the prior (Girshick et al., 2011; Acerbi et al., 2014; Sprague et al., 2015). Numeric methods, while able to fit an arbitrary prior, are often slower and require hand-tuning of the numerical support and precision. Thus, while researchers have a varied toolkit for modeling the shapes of Bayesian priors, there is still a need to diversify our tools for using these models in perceptual research.

Our goal is to provide an overview of how Bayesian ideal observer models can be used in perceptual research, and to describe a computational approach that uses mixture of Gaussian models to flexibly and efficiently model the influence of priors on perception. First, we review the general mathematical principles that link a Bayesian ideal observer to psychophysical data. Then, we present the analytic solutions for psychophysical quantities assuming a simple Gaussian prior and Gaussian measurement noise. Next, we introduce a mixture of Gaussians model of priors that provides increased flexibility. Mixture of Gaussian priors have been employed in other contexts, such as computer vision and signal processing (Olshausen and Millman, 1999; Snoussi and Mohammad-Djafari, 2001), but are not commonly used in ideal observer models (but see related applications for modeling perceptual inferences by Acerbi et al., 2014; Orhan and Jacobs, 2014). Lastly, we introduce a new analytical approximation that increases the computational efficiency of the mixture of Gaussians model. This approximation offers improvements in efficiency for adaptive experimental methods (e.g., adaptive stimulus staircasing) as compared with fully numerical approaches. An accompanying MATLAB (MathWorks) toolkit provides implementations that can be used to simulate and fit psychophysical data.

## Materials and Methods

### Bayesian ideal observer models

In a Bayesian ideal observer model, the observer makes a noisy measurement *m* of a stimulus *x* and uses that measurement to generate an estimate of the stimulus in the world $\hat{x}$ or to select an appropriate behavioral response *r*. We can represent this mapping of measurement onto response with the function *r *=* T*(·) where *T* is some estimation function. For example, in the context of a psychophysical experiment, *T*(·) may represent a point estimate of the presented stimulus (in which case $r=T(m)=\hat{x}$) or a binary judgment in a two-alternative forced-choice (2AFC) experiment (e.g., $r=T(m_{1},m_{2})=$ “yes” when queried whether $x_{2}>x_{1}$).

A Bayesian ideal observer selects the optimal response to a set of stimuli on the basis of the three components:

- a prior distribution *p*(*x*)
- a likelihood *p*(*m*|*x*)
- a loss function *L*(*x*, *r*)

The prior *p(x)* represents the observer’s knowledge of the probability of encountering the stimulus based on previous experience. The likelihood p(m|x), the probability of a measurement given the stimulus, captures the noisiness in the observer’s measurement of the stimulus. The noisiness depends on both external factors (such as signal strength and presentation time) and internal factors (such as neuronal noise and attentional state).

To obtain the observer’s belief about the current stimulus *x* given a measurement *m* we first use Bayes’ rule to obtain the posterior distribution, p(x|m), as follows:

(1) $$p(x|m)=\frac{p(m|x)p(x)}{p(m)}.$$

**Figure 1.**
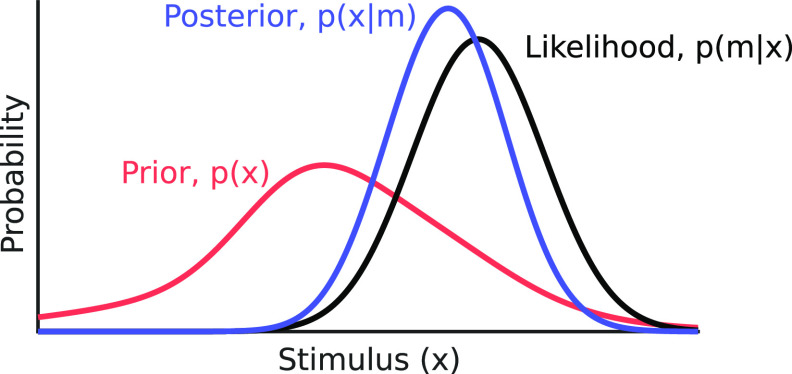
Canonical Bayesian computation. This figure illustrates Bayes’ rule, by which a posterior is the product of a prior (the observer’s knowledge of the probability of encountering the stimulus) and a likelihood (the set of stimulus values associated with a given a measurement). The posterior is scaled by the inverse of the marginal likelihood. Toolkit script: Fig1_BayesianDemo.m.

The posterior represents the probability distribution of a stimulus, given the current measurement, and can thus be used for drawing inferences. Here, *p*(*m*) is the model evidence (or marginal likelihood) that serves to normalize the posterior. This calculation is represented graphically in Figure 1. Since *p*(*m*) is a scalar value and does not affect the shape of the posterior, we can note that p(x|m)∝p(m|x)p(x).

**Figure 2.**
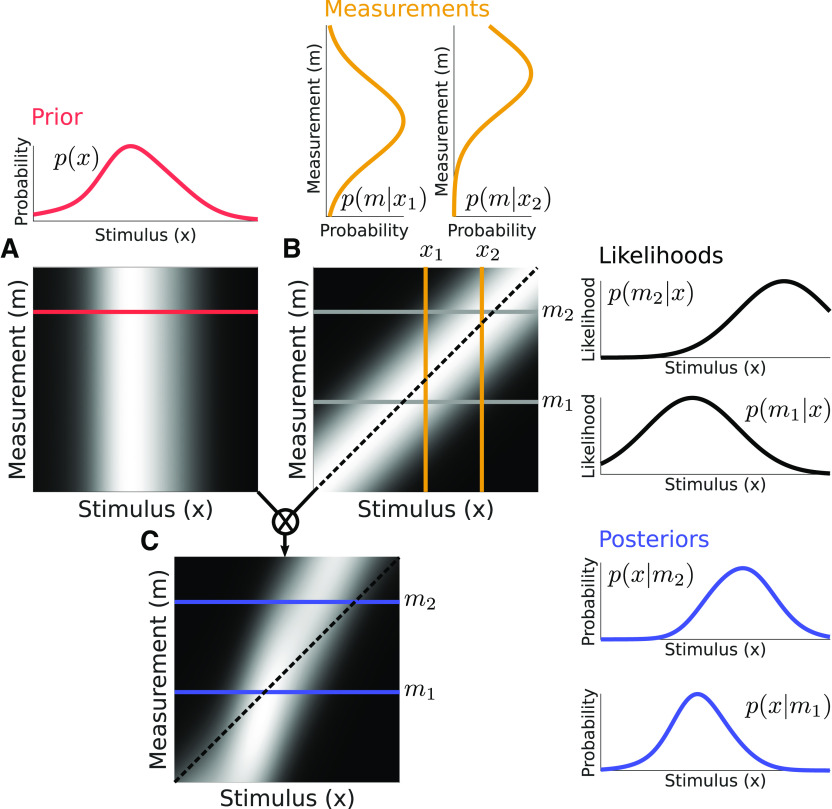
The canonical Bayesian computation as in Figure 1 but expanded to a set of likelihood functions. The prior (***A***) is multiplied by the likelihood defined by a given measurement (***B***, shown for m_1_ and m_2_) to obtain the posterior (***C***). Note that the shape of the posteriors change for different likelihoods since the prior is non-Gaussian, but the posteriors are overall drawn to the largest probability region of the prior. In each panel, the heat map values represent probability with higher intensity mapping to higher probability. Identity lines are indicated with dashed black lines. Toolkit script: Fig2_2DBayesianDemo.m.

This simple illustration, however, shows a likelihood based on only one example measurement. If we instead consider the full range of possible measurements, as shown in Figure 2, we can see how the resulting shape of the posterior varies. Figure 2*A* shows the prior as a function of *x*. By definition, the prior is independent of the measurement *m*, so it varies horizontally, but is constant along the vertical dimension. This two-dimensional (2D) format, similar to that used in (Girshick et al., 2011), helps illustrate the point that the posterior (Fig. 2*C*) arises from pointwise multiplying the prior (Fig. 2*A*) and likelihood (Fig. 2*B*). Figure 2*B* illustrates the likelihood by plotting the probability of the observer making each measurement, conditioned on each possible stimulus value. This 2D distribution is generated by assuming that the measurement associated with each stimulus value is corrupted by additive Gaussian noise, but is unbiased. A vertical slice through B represents what we refer to as the measurement distribution $p(m|x_{n})$, which is the probability over measurement values *m* given a particular stimulus *x_n_*. A horizontal slice through B, on the other hand, represents the likelihood function *p*(*m_n_*,*x*), which is the probability of a given measurement *m* as a function of different stimulus values *x*. Thus, the 2D object p(m|x) may represent either the likelihood when it is conditioned on a specific measurement and considered as a function of the stimulus, or a measurement distribution when it is conditioned on a specific stimulus and considered as a function of measurements.

While the likelihood is the pertinent quantity for applying Bayes’ rule, the measurement distribution is the relevant quantity when considering samples of the noisy sensory observation process. Note the measurement distribution is a true probability density function based on a noise model (here, we use additive Gaussian noise). The likelihood, on the other hand, is not generally a probability distribution because it does not necessarily integrate to one.

By multiplying each row of the prior and likelihood plots and normalizing, we obtain the set of possible posterior distributions p(x|m) for each possible measurement (Fig. 2*C*). Note that since the prior is non-Gaussian and steeper around the left flank of the peak, the posteriors are more concentrated around these values.

Finally, a loss function is needed to complete the model. The loss function *L*(*x*,*r*) refers to the penalty of making a response *r* when the true stimulus was *x*. An optimal decision rule is one where the observer will minimize the loss on average over the course of a set of responses. To calculate the expected loss of a particular response, we can find the expected loss under the posterior:

(2) E[L(x,r)]=∫L(x,r)p(x|m)dx.

A decision rule is Bayes optimal under a particular loss function if it minimizes the expected loss for all measurements. That is, T*(·) is Bayes optimal if for all estimation functions *T*(*m*) and all measurement values *m*:

(3) $$E[L(x,T^{*}(m))]\leq E[L(x,T(m))].$$

Note that here we show *T*(·) as a function of a single *m*, but it may also take multiple measurements into account, as in a two-alternative forced-choice paradigm (2AFC). In the following sections, we will discuss this loss in more concrete terms in the context of point estimation and 2AFC tasks.

While the derivations outlined in this paper do not assume any particular stimulus, they do assume that the measurements are unbiased, and that the measurement noise is additive and Gaussian distributed. In this case, the likelihood always takes the form of a Gaussian. Under these assumptions, the mean of the likelihood varies with and is equal to the measurement. We also assume that the width of the likelihood (i.e., the amount of noise) does not inherently vary with the measurement. However, the Weber–Fechner law across many stimuli suggests that this assumption does not hold if stimulus values are represented in many common sense units (e.g., candelas per square meter for luminance, visual degrees per second for speed), because sensory thresholds in these units increase systematically as stimulus values increase (Hecht, 1924; McKee et al., 1986; Pardo-Vazquez et al., 2019). Thus, a transformation of the stimulus values from physical space to “sensory space” may often be necessary to satisfy this assumption (Stocker and Simoncelli, 2006; Kwon et al., 2015). Indeed, the Weber–Fechner law suggests that the width of the likelihood or measurement distribution is approximately constant in logarithmic units across many stimulus domains (although deviations have been noted). For example, if one were to model visual speed perception, the measurement distribution and prior could be represented in terms of p(log(m)|log(x)) and *p*(*x*), respectively. Throughout this document, we will represent the likelihoods as Gaussians even if a transformation is necessary, to keep the estimation of the prior computationally tractable. For reference, Table 1 provides a summary of notation used for each of the ideal observer parameters.

**Table 1:** General notation

Value	Notation
Stimulus value	*x*
Sensory measurement	*m*
Stimulus estimate	$\hat{x}$
Response	*r*
Likelihood SD	σ
Prior mean, SD	ν, γ
Posterior mean, SD	μ*_post_*, *σ_post_*

### Modeling psychophysical data from an observer with a Gaussian prior

We begin with a simple case in which the prior takes the form of a Gaussian distribution. If this condition is met, the posterior has an analytic solution and is also Gaussian. This property follows from the general rule defining the product of any two Gaussians. Specifically, if we denote a Gaussian distribution generally as $N(a,b^{2})$ with mean *a* and standard deviation *b*, we can write the prior as $N(\nu ,\gamma^{2})$ (see Table 1). We define the likelihood as a Gaussian function with its mean equal to the measurement value *m* and a SD of *σ*: $N(m,\sigma^{2})$. We can then write the posterior as:

(4) $$\begin{array}{l} p(x|m)=\frac{1}{\rho }N(m,\sigma^{2})N(\nu ,\gamma^{2})\\ =N(\mu_{post},\sigma_{post}^{2}) \end{array}$$

where the normalizing constant *ρ*, which relates the posterior to the product of prior and likelihood, is given by:

(5) $$\rho =\frac{1}{\sqrt{2\pi }}\left(\frac{\sigma_{post}}{\sigma \gamma }\right)\exp\left[-\frac{m^{2}}{2\sigma^{2}}-\frac{\nu^{2}}{2\gamma^{2}}+\frac{\mu_{post}^{2}}{2\sigma_{post}^{2}}\right],$$

and the posterior variance and mean are given by:

(6) $$\sigma_{post}^{2}=\sigma^{2}\left(\frac{\gamma^{2}}{\sigma^{2}+\gamma^{2}}\right)$$

(7) $$\mu_{post}=m\left(\frac{\gamma^{2}}{\sigma^{2}+\gamma^{2}}\right)+\nu \left(\frac{\sigma^{2}}{\sigma^{2}+\gamma^{2}}\right).$$

#### Selecting a sensory estimate from the posterior

To start linking this framework to psychophysical data, we first consider an experiment in which we want to fit a Bayesian ideal observer model to a set of data in which participants reported point estimates of the presented stimuli (e.g., through method of adjustment such that x′=r is a possible estimate response when *x* is the true value). To convert the posterior into an optimal estimate, we can assert a loss function for our Bayesian ideal observer. In the general form, this loss function will determine the Bayes estimate that minimizes the expected error defined in Equation 2:

(8) $$\hat{x}=\underset{x\prime }{\text{argmin}}\int L(x,x\prime )p(x|m)dx.$$

Two commonly used loss functions are the zero-one loss (where the loss is 0 when (x−x′)=0, and 1 for all other values), and squared error loss ( $L(x,x\prime )={\left(x-x\prime \right)}^{2}$). Using zero-one loss, we obtain a Bayes optimal estimate $\hat{x}$ that is the mode of the posterior, the maximum a posteriori (MAP) estimate:

(9) $${\hat{x}}_{MAP}=\underset{x}{\text{argmax}}p(x|m).$$

For an ideal observer that uses a squared error loss function, the Bayesian least squares (BLS) estimate is the mean of the posterior:

(10) $${\hat{x}}_{BLS}=E[x|m].$$

When the posterior is Gaussian, the MAP and BLS estimates are equivalent and equal to μ_post_ (Eq. 7), which can be simplified to:

(11) $${\hat{x}}_{BLS}={\hat{x}}_{MAP}=\alpha m+\tilde{\nu }.$$

Here, we have simplified the equation for μ_post_ such that α is a shrinkage factor that determines how biased the posterior is toward the prior mean:

(12) $$\alpha =\left(\frac{\gamma^{2}}{\gamma^{2}+\sigma^{2}}\right)$$

and $\tilde{\nu }$ offsets the posterior when the prior is not zero-centered:

(13) $$\tilde{\nu }=\left(\frac{\sigma^{2}}{\sigma^{2}+\gamma^{2}}\right)\nu .$$

With these simplifications we can rewrite the posterior as:

(14) $$p(x|m)=N(\alpha m+\tilde{\nu },\alpha \sigma^{2}).$$

When ${\hat{x}}_{BLS}={\hat{x}}_{MAP}$, we simply adopt $\hat{x}$ to denote the estimate. The solution for $\hat{x}$ here can also be considered as a weighted average of the prior and likelihood means, where the weights are inversely proportional to the variance of the prior and likelihoods (Landy et al., 1995). To make that link explicit, we can represent Equation 11 as $\hat{x}=\alpha m+(1-\alpha )\nu$, since $\tilde{\nu }$ is equal to (1 – *α*)*ν*. Note that when the posterior is not Gaussian, the MAP and BLS estimates are not necessarily equivalent.

#### Distribution of sensory estimates

**Figure 3.**
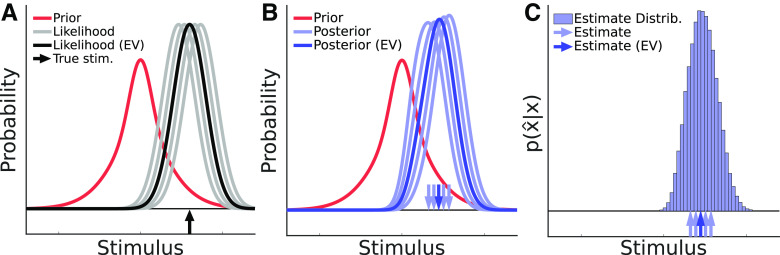
The distribution of sensory estimates arises from the variability in the measurement values about the expected value across trials (EV; i.e., the true stimulus value). ***A***, On a given trial, a likelihood is defined around the observed measurement. Here, we plot the expected value of this likelihood for a given true stimulus, as well as other possible likelihoods that occur on a set of trials. The prior is shown for reference. The upward arrow indicates the true stimulus that used to generate the likelihood. ***B***, The resulting posteriors for each trial are shown, along with downward arrows indicating the estimates ( $\left\{\hat{x}\right\}$) derived from these posteriors. ***C***, Over many trials, these estimates (now indicated as upward arrows) create an estimate distribution, which can be predicted for a Bayesian ideal observer with a given prior and amount of sensory noise. When the prior is Gaussian, there is a closed form expression for this distribution. Toolkit script: Fig3_EstimateDistribution.m.

While the ideal observer model outlined in this paper is defined from the perspective of the observer, we will briefly shift our perspective to that of an experimenter to demonstrate how the model can be used in practice. In a task in which the observer is making repeated point estimates of the stimulus (e.g., judging its visual brightness, auditory volume, or speed), the mean of the measurement distribution on each trial will be equal to the true value of the stimulus, *x*, and we can define $T(m)=\hat{x}=\alpha m+\tilde{\nu }$ as the function by which the ideal observer converts noisy measurements into a response on each trial. While this is a deterministic function, the value will vary from one trial to another because of variability in the measurement *m*. The responses thus form an estimate distribution $p(\hat{x}|x)$, the probability distribution of estimates, given a particular stimulus (Fig. 3).

If we want to infer the underlying ideal observer parameters from a set of real behavioral data, we can fit a set of empirically measured observer estimates to this estimate distribution. To do so, we define an analytic form of this estimate distribution $p(\hat{x}|x)$ with a substitution of variables in which we substitute $T^{-1}(\hat{x})$ for *m* in the measurement distribution $p(m|x)=N(x,\sigma^{2})$. First, we solve for $T^{-1}(\hat{x})$ and the first derivative of this function with respect to $\hat{x}$:

(15) $$T^{-1}(\hat{x})=m=\frac{\hat{x}-\tilde{\nu }}{\alpha }$$

(16) $$\frac{d}{d\hat{x}}T^{-1}(\hat{x})=\frac{1}{\alpha },$$

and then perform the substitution of variables:

(17) $$\begin{array}{l} p(\hat{x}|x)=\frac{1}{\sqrt{2\pi \sigma^{2}}}\exp\left[-\frac{{\left(T^{-1}(\hat{x})-x\right)}^{2}}{2\sigma^{2}}\right]\left|\frac{dT^{-1}(\hat{x})}{d\hat{x}}\right|\\ =\frac{1}{\sqrt{2\pi \sigma^{2}}}\exp\left[-\frac{{\left(\frac{\hat{x}-\tilde{\nu }}{\alpha }-x\right)}^{2}}{2\sigma^{2}}\right]\left|\frac{1}{\alpha }\right|\\ =\frac{1}{\sqrt{2\pi \alpha^{2}\sigma^{2}}}\exp\left[-\frac{{\left(\hat{x}-(\alpha x+\tilde{\nu })\right)}^{2}}{2\alpha^{2}\sigma^{2}}\right] \end{array}$$

which we can denote simply as:

(18) $$p(\hat{x}|x)=N(\alpha x+\tilde{\nu },\alpha^{2}\sigma^{2}).$$

While we could also derive the estimate distribution more simply using the identity for the affine transformation of Gaussian random variables, we use a substitution of variables here to draw a parallel to the mixture of Gaussians case in the next section. Note that the form of the estimate distribution is similar to the posterior distribution associated with a single measurement (Eq. 14) with two key differences: the mean of the estimate distribution is dependent on the stimulus x instead of any specific noisy measurement, and the variance is equal to the variance of the likelihood scaled by α^2^ instead of α.

This distribution of observer estimates, given the stimulus, provides the likelihood function for fitting the Bayesian ideal observer model to data by performing maximum likelihood estimation (MLE; not to be confused with the likelihood of a Bayesian observer). Specifically, it is a likelihood when considered as a function of the model parameters θ = { ν, γ, σ }. Given a set of paired stimuli and observer reports ${\left\{(x_{t},{\hat{x}}_{t})\right\}}_{t}^{N}=1$ from a set of conditionally independent trials t = 1,…,N, the model likelihood is given by:

(19) $$p(\{\hat{x_{t}}\}|\{x_{t}\},\theta )=\prod_{t=1}^{N}\frac{1}{\sqrt{2\pi \alpha^{2}\sigma^{2}}}\;\exp\left[-\frac{{\left({\hat{x}}_{t}-(\alpha x_{t}+\tilde{\nu })\right)}^{2}}{2\alpha^{2}\sigma^{2}}\right].$$

In practice, we optimize θ by minimizing the negative log-likelihood, which is obtained by taking the negative log of this expression:

(20) $$\begin{array}{l} -\log\;[p(\{{\hat{x}}_{t}\}|\{x_{t}\},\theta )]=-\left[\sum_{t=1}^{N}\log\left(\frac{1}{\sqrt{2\pi \alpha^{2}\sigma^{2}}}\right)-\left(\frac{{\left(\hat{x}-(\alpha x_{t}+\tilde{\nu })\right)}^{2}}{2\alpha^{2}\sigma^{2}}\right)\right]\\ =\frac{N}{2}\log(2\pi \alpha^{2}\sigma^{2})+\frac{1}{2\alpha^{2}\sigma^{2}}\sum_{t=1}^{N}({\hat{x}}_{t}-(\alpha x_{t}+\tilde{\nu }){)}^{2}. \end{array}$$

#### Two-alternative forced-choice task

Experimenters often avoid having research participants report point estimates of stimuli because the origin of the noise in the measurement is ambiguous. For example, responses that incorporate a motor component may be contaminated by motor noise in addition to sensory noise. To avoid this issue, participants can make a categorical judgment about stimuli in perceptual space that can be related back to physical qualities of the stimulus. One such paradigm is a two-alternative forced-choice (2AFC) task in which participants view two stimuli either sequentially or concurrently and must select which of the two best fits the instructions they are given. In a speed judgment task, for example, the instruction might be: “indicate which of the two stimuli appeared to move faster”. Often, this task is repeated for a range of stimulus values, such as stimulus speed, to build up a psychometric function. This function, for example, might describe the probability that a test stimulus is perceived as moving faster than a fixed reference stimulus, as a function of the test stimulus speed. Importantly, the two stimuli should differ in reliability to estimate the best fitting parameters for both the likelihood and the prior.

**Figure 4.**
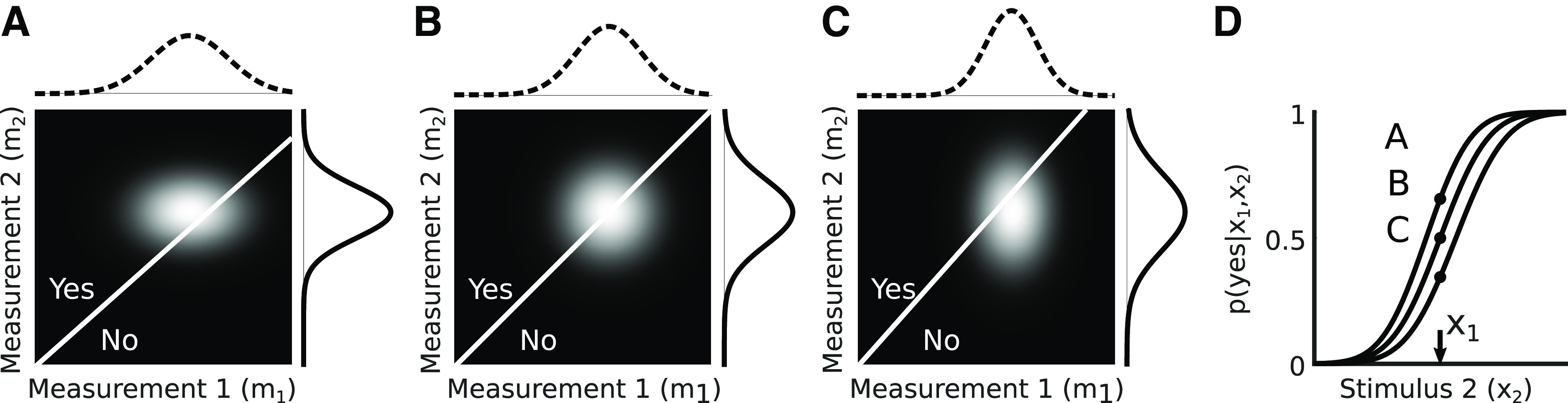
Graphical illustration of computing the observer’s psychometric curve for a 2AFC task. ***A***, Computing a single point on the psychometric curve when $x_{1}=x_{2}=3$ for measurement noise variances $\sigma_{1}^{2}=0.75,\;\sigma_{2}^{2}=0.5$ and a prior with v = 0 and γ = 1.5. Dashed line (top) shows the measurement distribution $p(m_{1}|x_{1})$ and solid line (right) shows measurements distribution *p*(*m|x*_2_). The 2D grayscale image shows the joint distribution of observer measurements given the stimuli *x*_1_ and *x*_2_, formed by the product of the two measurement distributions along the top and right. The white diagonal line is the observer’s decision boundary, corresponding to measurement values for which the inferred speeds are equal. The probability that the observer reports “yes” (i.e., that x_2_ exceeded x_1_) is the area above the decision boundary (point “A” in panel ***D***). ***B***, Same as panel ***A*** but with equal noise variances $\sigma_{m1}^{2}=\sigma_{m2}^{2}=0.64$. ***C***, Same as panel ***A*** but with noise variances $\sigma_{m1}^{2}=0.5,\;\sigma_{m2}^{2}=0.75$. ***D***, Full psychometric curves for the noise variances used in panels ***A–C***, showing the probability that the observer reports “yes” as a function of the stimulus *x*_2_. The points labeled A, B, C represent the sum of the probability above the diagonal in panels ***A–C***.

If we consider two stimuli *x*_1_ and *x*_2_, on each trial, the observer makes two noise-corrupted measurements, which we model with two measurement distributions $p(m_{1}|x_{1})$ and $p(m_{2}|x_{2})$ or a single joint distribution $p(m_{1},m_{2}|x_{1},x_{2})$ (see Fig. 4 for examples). The ideal observer selects an optimal response *r* based on a decision function that takes both measurements as input ( $T(m_{1},m_{2})$). Here, we assume this function indicates whether or not stimulus *x*_2_ best satisfies the instructions given the measurements (e.g., in our speed judgment example, was *x*_2_ faster than *x*_1_). This is defined by the following decision rule:

(21) $$r=T(m_{1},m_{2})=\{\begin{array}{ll} 1 & p(x_{2}>x_{1}|m_{1},m_{2})>0.5\\ 0 & \text{otherwise} \end{array},$$

where $p(x_{2}>x_{1}|m_{1},m_{2})$ is determined for each pair $\left(m_{1},m_{2}\right)$ by:

(22) $$p(x_{2}>x_{1}|m_{1},m_{2})=\int_{-\infty }^{\infty }\int_{x_{1}}^{\infty }p(x_{1},x_{2}|m_{1},m_{2})dx_{2}dx_{1}.$$

Since we model the likelihoods as independent and the posteriors are both Gaussian (at this point in the derivations), we can more succinctly say this occurs whenever the estimate ${\hat{x}}_{2}=\alpha_{2}m_{2}+{\tilde{\nu }}_{2}$ is greater than ${\hat{x}}_{1}=\alpha_{1}m_{1}+{\tilde{\nu }}_{1}$, which we can express using the decision rule:

(23) $$T(m_{1},m_{2})=\{\begin{array}{ll} 1 & \alpha_{2}m_{2}+{\tilde{\nu }}_{2}>\alpha_{1}m_{1}+{\tilde{\nu }}_{1}\\ 0 & \text{otherwise}. \end{array}$$

Because this is now a classification task, we adopt the loss function:

(24) $$L((x_{1},x_{2}),r)=|r-1(x_{2}>x_{1})|,$$

where 1(·) denotes an indicator function that evaluates to 1 when the input is true. For simplicity, we will represent the first case in Equation 23 as “yes” and the second case as “no”. Graphically, this equation is represented in Figure 4 as a white decision boundary in panels *A–C* for three different combinations of noise levels for m_1_ and m_2_. The slope of this line is determined by: $m_{2}=\frac{\alpha_{1}}{\alpha_{2}}m_{1}+\frac{{\tilde{\nu }}_{1}-{\tilde{\nu }}_{2}}{\alpha_{2}}$. If we want to solve for the probability of responding “yes” for a given *x*_2_ and *x*_1_ over repeated trials (i.e., a point on the psychometric curve), we can obtain a numerical solution by integrating the joint distribution above the decision boundary:

(25) $$p(“\text{yes}”\text{|}x_{\text{1}},x_{\text{2}})=\int_{-\infty }^{\infty }\int_{-\infty }^{\infty }T(m_{1},m_{2})p(m_{1}|x_{1})p(m_{2}|x_{2})dm_{1}dm_{2}.$$

The results of this integration for Figure 4*A–C* are shown in Figure 4*D*, along with the full psychometric curves.

However, Bayesian ideal observer models with Gaussian posteriors also allow for an equivalent analytical alternative to this calculation. Specifically, we can obtain an analytic solution for points on the psychometric curve via an alternative model of the Bayesian observer in which the observer computes the MAP estimate for each stimulus and then compares which of the two is larger. This method has been used previously (Stocker and Simoncelli, 2006) and is equivalent to the optimal computation in Equation 25 when the prior and likelihoods are both Gaussian. Since ${\hat{x}}_{MAP}={\hat{x}}_{BLS}$, this solution works for both estimators. The probability that a given estimate of *x*_2_ ( ${\hat{x}}_{2}$) is greater than the estimate *x*_1_ ( ${\hat{x}}_{1}$) can be obtained by integrating over the estimate distributions for the two stimuli in what is essentially a signal detection problem (Green and Swets, 1966):

(26) $$p(“\text{yes}”\text{|}x_{\text{1}},x_{\text{2}})=\int_{-\infty }^{\infty }\int_{-\infty }^{{\hat{x}}_{2}}p({\hat{x}}_{2}|x_{2})p({\hat{x}}_{1}|x_{1})d{\hat{x}}_{1}d{\hat{x}}_{2}.$$

Equivalently, $p(“\text{yes}”{\text{|x}}_{\text{1}},{\text{x}}_{\text{2}})$ can be expressed as the integral over positive values of ${\hat{x}}_{2}-{\hat{x}}_{1}$ in the probability distribution $p({\hat{x}}_{2}-{\hat{x}}_{1}|x_{1},x_{2})$. This has an analytic solution since the difference of two Gaussian random variables is itself a Gaussian. For a Gaussian prior, the estimate distributions are indeed Gaussian (see Eq. 18) so this difference $p({\hat{x}}_{2}-{\hat{x}}_{1}|x_{1},x_{2})$ is defined as:

(27) $$\begin{array}{l} p({\hat{x}}_{2}-{\hat{x}}_{1}|x_{1},x_{2})=N(\alpha_{2}x_{2}+{\tilde{\nu }}_{2},\alpha_{2}^{2}\sigma_{2}^{2})-N(\alpha_{1}x_{1}+{\tilde{\nu }}_{1},\alpha_{1}^{2}\sigma_{1}^{2})\\ =N(\alpha_{2}x_{2}-\alpha_{1}x_{1}+{\tilde{\nu }}_{2}-{\tilde{\nu }}_{1},\alpha_{2}^{2}\sigma_{2}^{2}+\alpha_{1}^{2}\sigma_{1}^{2}) \end{array}.$$

From this equation, $p(“\text{yes}”\text{|}x_{\text{1}},x_{\text{2}})$ can be attained simply by integrating over positive values of this difference:

(28) $$p(“\text{yes}”\text{|}x_{\text{1}},x_{\text{2}})=\int_{0}^{\infty }N(\alpha_{2}x_{2}-\alpha_{1}x_{1},\alpha_{2}^{2}\sigma_{2}^{2}+\alpha_{1}^{2}\sigma_{1}^{2}).$$

To simplify the calculation of this integral, we can convert the difference distribution to a standard normal ϕ(·) by subtracting the mean and scaling all values by the inverse of the SD. The location on the standard normal curve that corresponds to the lower bound on the integral in Equation 28 is then equal to the original mean divided by the SD. This is useful because it allows us to integrate the standard normal above this (standardized) mean to find $p(“\text{yes}”\text{|}x_{\text{1}},x_{\text{2}})$ for a given *x*_2_. That is, instead of integrating the original normal from zero to infinity, we now integrate the standard normal up to the standardized mean. Lastly, we take advantage of the fact that the standard normal is symmetric about its mean to write the equation as follows:

(29) $$p(“\text{yes}”\text{|}x_{\text{1}},x_{\text{2}})=\Phi \left(\frac{\alpha_{2}x_{2}-\alpha_{1}x_{1}}{\sqrt{\alpha_{2}^{2}\sigma_{2}^{2}+\alpha_{1}^{2}\sigma_{1}^{2}}}\right),$$

where Φ(·) is the cumulative standard normal:

(30) $$\Phi (K)=\frac{1}{\sqrt{2\pi }}\int_{-\infty }^{K}\exp[\;\frac{-t^{2}}{2}]dt,$$

and the symmetry about the mean of ϕ indicates that $\int_{-K}^{\infty }\phi (t)=\int_{-\infty }^{K}\phi (t)=\Phi (K)$ for all values of *K*.

We can again take the perspective of the experimenter to demonstrate how to fit the ideal observer model to 2AFC data. This analytic solution is an efficient way to estimate the underlying parameters of the Bayesian ideal observer model given a dataset ${\left\{x_{1,t},x_{2,t},T_{t}\right\}}_{t=1}^{N}$, where *T_t_* is the participant’s response to stimulus pair $x_{1,t},x_{2,t}$ on trial *t*. As in the point estimate case, we can solve for the best fitting parameters θ = {*v*, γ, σ_1_, σ_2_} with MLE in which we minimize the following negative log-likelihood function:

(31) $$-\log\;[p(\{T\}|\{x_{1},x_{2}\},\theta )]=-\sum_{t=1}^{N}T_{t}\log\;[p(“\text{yes}”\text{|}x_{\text{1},t},x_{\text{2},t})]\text{+ }(\text{1}–T_{t})\log[\text{1}–p(“\text{yes}”\text{|}x_{\text{1},t},{\text{x}}_{\text{2},t})].$$

#### Summary

Up to this point, we have described how to determine the posterior, the individual sensory estimates, the sensory estimate distribution, and the results of a 2AFC task for a Bayesian ideal observer with a Gaussian prior and likelihood. In the next section, we will generalize this framework by deriving the same quantities for an observer with a prior that can be modelled more flexibly as a mixture of Gaussian components.

### Modeling psychophysical data for an observer with a mixture of Gaussians prior

While the approach outlined in the previous section is computationally efficient, it assumes that the observer’s prior is well fit by a single Gaussian. This is unlikely to be the case assuming that the prior reflects knowledge of natural scene statistics, since many physical quantities have much heavier tails than a Gaussian (Dong and Atick, 1995; Sprague et al., 2015) or are even multimodal (Girshick et al., 2011; Kim and Burge, 2018). Accurately modeling these shapes is important. For example, long-tailed priors would predict that biases are reduced for stimulus values that fall within the the flatter regions of the stimulus probability distribution than in the more peaked regions. In this section, we propose an approach based on a mixture of Gaussians that retains some of the efficiency of the single Gaussian prior while better approximating realistic priors. Table 2 lists a summary of the additional notation adopted for this section.

**Table 2:** Mixture of Gaussians notation

Value	Notation
Weight (prior component i)	*w*_i_
Mean (prior component i)	ν_i_
SD (prior component i)	γ_i_

**Figure 5.**
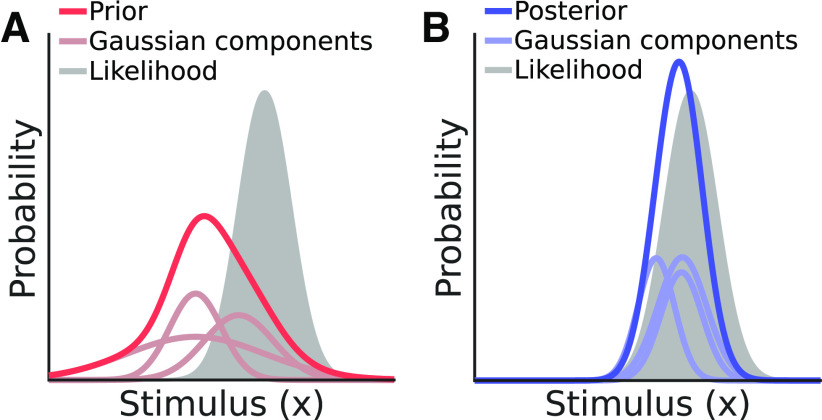
Prior and posterior defined by a mixture of Gaussian components. ***A***, The prior of a Bayesian observer (dark red line) can be modeled as a mixture of Gaussian components (light red lines). ***B***, When combined with a Gaussian likelihood, the resulting posterior is also a mixture of Gaussians. Similar to the posterior resulting from a single Gaussian prior, the mixture of Gaussians posterior is biased relative to the likelihood. Likelihoods are shaded here for visual clarity. Toolkit script: Fig5_MoGprior.m.

Consider an observer with a prior defined by a mixture of *C* Gaussian components:

(32) $$p(x)=\sum_{i=1}^{C}w_{i}N(\nu_{i},\gamma_{i}^{2}),$$

where *w_i_* ≥ 0 is the weight of the *i*th component, with $\sum w_{i}=1$, and ν_i_ and $\gamma_{i}^{2}$ are the mean and variance of the *i*th Gaussian component, respectively (Fig. 5*A*, red lines). If we assume a Gaussian likelihood with variance σ^2^, the posterior is also a mixture of Gaussians (Fig. 5*B*, blue lines):

(33) $$p(x|m)=\sum_{i=1}^{C}{\tilde{w}}_{i}(m)N(\alpha_{i}m+{\tilde{\nu }}_{i},\alpha_{i}\sigma^{2}),$$

where α_i_ and ${\tilde{\nu }}_{i}$ are the shrinkage factor and mean of the *i*th posterior component, respectively:

(34) $$\alpha_{i}=\frac{\gamma_{i}^{2}}{\gamma_{i}^{2}+\sigma^{2}}$$

(35) $${\tilde{\nu }}_{i}=\left(\frac{\sigma^{2}}{\sigma^{2}+\gamma_{i}^{2}}\right)\nu_{i}.$$

This is the mixture of Gaussians version of the posterior given in Equation 14. Here, ${\tilde{w}}_{i}(m)$ is a set of adjusted weights that combine the weights *w_i_* of the individual components of the prior, the scale factors *ρ_i_*(*m*) for each of the components of the posterior (analogous to Eq. 5), and a normalization step to ensure the weights all sum to 1. To determine ${\tilde{w}}_{i}(m)$, we can first define each *ρ_i_*(*m*) as:

(36) $$\rho_{i}(m)=\frac{1}{\sqrt{2\pi }\sqrt{\gamma_{i}^{2}+\sigma^{2}}}\exp\left[-\frac{m^{2}}{2\sigma^{2}}-\frac{\nu_{i}^{2}}{2\gamma_{i}^{2}}+\frac{{\left({\tilde{\nu }}_{i}+\alpha_{i}m\right)}^{2}}{2\alpha_{i}\sigma^{2}}\right],$$

and by substituting for ${\tilde{\nu }}_{i}$ and α_i_ with Equations 35 and 34, respectively, then simplifying, we obtain:

(37) $$\rho_{i}(m)=\frac{1}{\sqrt{2\pi }\sqrt{\gamma_{i}^{2}+\sigma^{2}}}\exp\left[-\frac{{\left(m-\nu_{i}\right)}^{2}}{2(\gamma_{i}^{2}+\sigma^{2})}\right].$$

Note that *ρ_i_*(*m*) is inversely related to the difference between the measurement *m* and the prior component mean *ν_i_*. Therefore, the posterior shape will change relative to the likelihood, not just shift as in the single Gaussian prior case. That is, as the measurement changes, the relative weight of each component changes. We can combine the scaling effects of *w_i_* and *ρ_i_*(*m*) to define:

(38) $$v_{i}(m)=w_{i}\rho_{i}(m)=\frac{w_{i}}{\sqrt{\gamma_{i}^{2}+\sigma^{2}}}\phi \left(\frac{m-\nu_{i}}{\sqrt{\gamma_{i}^{2}+\sigma^{2}}}\right),$$

which is then normalized by the sum of all *v_i_* to obtain the set of adjusted weights ${\tilde{w}}_{i}(m)$:

(39) $${\tilde{w}}_{i}(m)=\frac{v_{i}(m)}{\sum_{i=1}^{C}v_{i}(m)}.$$

In the following sections, we will first demonstrate how to fit the mixture of Gaussians prior to point estimation and 2AFC data using numerical evaluation of the log-likelihood. We then derive an analytical approximation that can reduce the computational load necessary to estimate the observer parameters.

#### Selecting a sensory estimate from the posterior

As before, let us first consider the case where we want to estimate a participant’s prior from a set of point estimates from an experimental dataset. We can use the posterior derived in Equation 33 and an appropriate loss function to define an optimal estimate $\hat{x}$. For the mixture of Gaussians posterior, the MAP and BLS estimates differ. Here, we will consider only ${\hat{x}}_{BLS}$, since this estimate has an analytical solution in the mean of the posterior:

(40) $${\hat{x}}_{BLS}=\sum_{i=1}^{C}{\tilde{w}}_{i}(m)(\alpha_{i}m+{\tilde{\nu }}_{i}).$$

Without an analytical solution ${\hat{x}}_{MAP}$ can be determined numerically and used instead in the numerical approaches described below. Note that if the posterior is multimodal, the BLS estimate may fall on a relatively unlikely value (since it is between the two modes of the posterior), and the MAP estimate may be unstable (since it may oscillate between the two modes depending on the measurement noise on a given stimulus presentation).

#### Distribution of sensory estimates

We can use Equation 40 to define $T(m)={\hat{x}}_{BLS}$ for the point estimation task. Unlike in the single Gaussian case, however, there is no clear analytic form for $T^{-1}({\hat{x}}_{BLS})$ with arbitrary mixture of Gaussians priors since ${\tilde{w}}_{i}$ is a function of *m*. To demonstrate this, consider a simplified form where all ${\tilde{\nu }}_{i}=0$ and it is clear that there is no way to solve for $T^{-1}({\hat{x}}_{BLS})$:

(41) $$\begin{array}{l} T(m)={\hat{x}}_{BLS}=m\sum_{i=1}^{C}{\tilde{w}}_{i}(m)\alpha_{i}\\ =m\frac{1}{\sum_{i=1}^{C}v_{i}(m)}\sum_{i=1}^{C}v_{i}(m)\alpha_{i} \end{array}.$$

Instead, we can numerically estimate $T^{-1}({\hat{x}}_{BLS})$ by first calculating $T(m)={\hat{x}}_{BLS}$ over a grid of points $\left\{{\hat{x}}_{BLS},m\right\}$ to create a look-up table to find {*m*} from $\left\{\hat{x}\right\}$ for a given set of Bayesian ideal observer parameters $\theta =\{w_{i},\nu_{i},\gamma_{i},\sigma \}$. With a goal of estimating an observer’s prior from a set of *N* point estimates (all with the same sensory noise level, σ), we can then evaluate the likelihood of the data given the putative model parameters, θ, using Equation 17:

(42) $$p(\{{\hat{x}}_{t}\}|\{x_{t}\},\theta )=\prod_{t=1}^{N}\frac{1}{\sqrt{2\pi \sigma^{2}}}\;\exp\left[-\frac{{\left(T^{-1}(\hat{x_{t}})-x_{t}\right)}^{2}}{2\sigma^{2}}\right]\left|\frac{dT^{-1}(\hat{x_{t}})}{d\hat{x_{t}}}\right|.$$

Note that we have abbreviated ${\hat{x}}_{BLS}$ to $\hat{x}$ for simplicity here. This process is then repeated for other parameter sets until we find an optimal solution that maximizes the likelihood of the data (or minimizes the negative log-likelihood). That is, finding θ that minimizes the following:

(43) $$\begin{array}{l} -\log\;[p(\{{\hat{x}}_{t}\}|\{x_{t}\},\theta )]=-\log[\prod_{t=1}^{N}\frac{1}{\sqrt{2\pi \sigma^{2}}}\;\exp\left[-\frac{{\left(T^{-1}(\hat{x_{t}})-x_{t}\right)}^{2}}{2\sigma^{2}}\right]\left|\frac{dT^{-1}(\hat{x_{t}})}{d\hat{x_{t}}}|\right]\\ =\sum_{t=1}^{N}\frac{{\left(x_{t}-T^{-1}({\hat{x}}_{t})\right)}^{2}}{2\sigma^{2}}+N\;\log[\sqrt{2\pi }\sigma ]-\sum_{t=1}^{N}\;\log\left|\frac{dT^{-1}({\hat{x}}_{t})}{d{\hat{x}}_{t}}\right|. \end{array}$$

The toolkit includes a function for this numerical approach (fitEstimData_numerical.m), which we will also return to in Results. This process can be computationally expensive, however, if we are trying to fit an observer’s prior with many Gaussian components (each of which is defined by three parameters *w*, ν, γ). While this may be acceptable for lower numbers of components and datasets that have already been collected, this is more problematic if the mixture of Gaussians model is used during the course of an experiment to guide an adaptive staircase.

To make the log-likelihood equation more tractable to solve, we can derive an approximate analytical solution for the point estimate distribution if we approximate Equation 40 using just the expected value of the measurement E(m)=x when calculating ${\tilde{w}}_{i}$:

(44) $$\begin{array}{l} {\tilde{w}}_{i}(x)\approx {\tilde{w}}_{i}(m) \end{array}$$

This approximation allows us to solve for *m* in Equation 41:

(45) $$\begin{array}{l} T(m)={\hat{x}}_{BLS}\approx \sum_{i=1}^{C}{\tilde{w}}_{i}(x)(\alpha_{i}m+{\tilde{\nu }}_{i}) \end{array}$$

(46) $$T(m)={\hat{x}}_{BLS}\approx m\sum_{i=1}^{C}{\tilde{w}}_{i}(x)\alpha_{i}+\sum_{i=1}^{C}{\tilde{w}}_{i}(x){\tilde{\nu }}_{i}.$$

We can then derive an analytic solution to $T^{-1}({\hat{x}}_{BLS})$ and its first derivative with respect to ${\hat{x}}_{BLS}$:

(47) $$T^{-1}({\hat{x}}_{BLS})=m\approx \frac{{\hat{x}}_{BLS}-\sum_{i=1}^{C}{\tilde{w}}_{i}(x)\nu_{i}}{\sum_{i=1}^{C}{\tilde{w}}_{i}(x)\alpha_{i}}$$

(48) $$\frac{d}{d{\hat{x}}_{BLS}}T^{-1}({\hat{x}}_{BLS})\approx \frac{1}{\sum_{i=1}^{C}{\tilde{w}}_{i}(x)\alpha_{i}},$$

and in turn use the substitution of variables to derive an (approximate) analytic solution in the form of a Gaussian:

(49) $$p({\hat{x}}_{BLS}|x)\approx \frac{1}{\sqrt{2\pi \sigma^{2}}}\exp\left[-\frac{{\left(T^{-1}({\hat{x}}_{BLS})-x\right)}^{2}}{2\sigma^{2}}\right]\left|\frac{dT^{-1}({\hat{x}}_{BLS})}{d{\hat{x}}_{BLS}}\right|p({\hat{x}}_{BLS}|x)\approx \frac{1}{\sqrt{2\pi \sigma^{2}}}\exp\left[-\frac{{\left(\frac{{\hat{x}}_{BLS}-\sum_{i=1}^{C}{\tilde{w}}_{i}(x)\nu_{i}}{\sum_{i=1}^{C}{\tilde{w}}_{i}(x)\alpha_{i}}-x\right)}^{2}}{2\sigma^{2}}\right]\left|\frac{1}{\sum_{i=1}^{C}{\tilde{w}}_{i}(x)\alpha_{i}}\right|$$

(50) $$p({\hat{x}}_{BLS}|x)\approx N\left(\sum_{i=1}^{C}{\tilde{w}}_{i}(x)(\alpha_{i}x+{\tilde{\nu }}_{i}),\sigma^{2}{\left(\sum_{i=1}^{C}{\tilde{w}}_{i}(x)\alpha_{i}\right)}^{2}\right).$$

This approximates the true estimate distribution with a Gaussian with a mean $\overset{C}{\underset{i=1}{\Sigma }}{\tilde{w}}_{i}(x)(\alpha_{i}x+{\tilde{\nu }}_{i})$ and variance $\sigma^{2}{\left(\overset{C}{\underset{i=1}{\Sigma }}{\tilde{w}}_{i}(x)\alpha_{i}\right)}^{2}$. Maximum likelihood estimation can then be used as described previously to find the model parameters that best explain an empirically measured estimate distribution. In Results, we analyze the regimes in which this is a good approximation.

#### Two-alternative forced-choice task

As with the point estimate distributions, we will again describe a numerical and approximate analytical approach for handling data from a 2AFC task.

**Figure 6.**
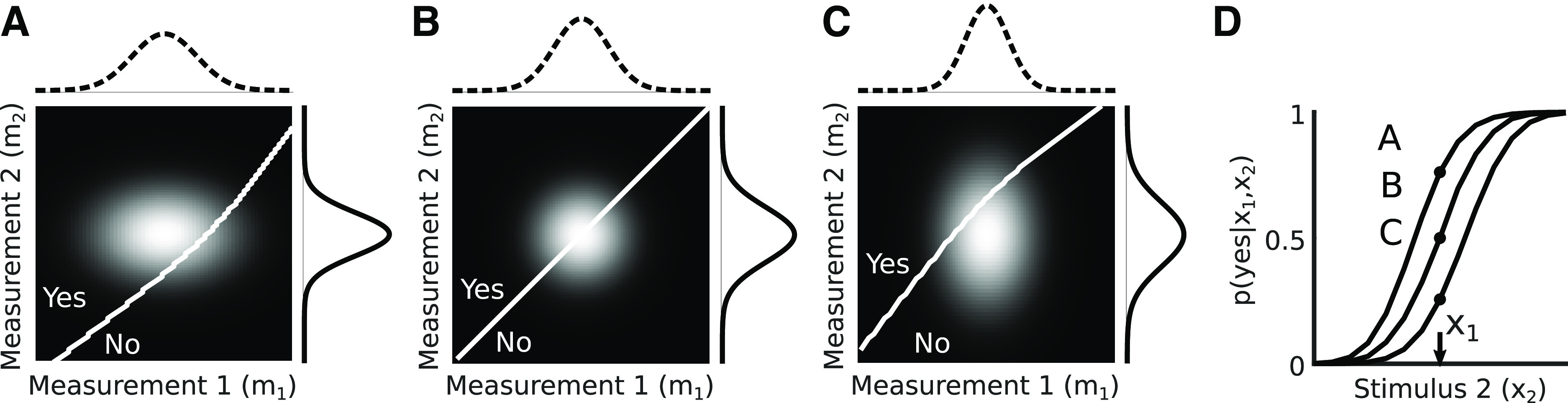
An extension of Figure 4 to a long-tailed prior defined by a mixture of Gaussians ( $\gamma_{1}=2,\gamma_{2}=0.6$ $\nu_{1}=\nu_{2}=0,\;w_{1}=w_{2}=0.5$), similar in appearance to the prior in Figure 7*A*. Here, the decision boundary representing $T(m_{1},m_{2})$ is nonlinear because the different components of the prior have different levels of influence on the percept as *m* varies. ***A***, As in Figure 4, the 2D grayscale image shows the joint distribution of the observer measurements given the stimuli *x*_1_ and *x*_2_, formed by the product of the two measurement distributions along the top and right. The white line is the observer's decision boundary. Here, *x*_1_ = *x*_2_ = 3 for measurement noise variances σ^2^_1_ = 0.75, σ^2^_1_ = 0.5. ***B***, Same as panel ***A***, but with equal noise variances σ^2^_m1_ = σ^2^_m2_ = 0.64. ***C***, Same as panel ***A***, but with measurement noise variances σ^2^_1_ = 0.75, σ^2^_1_ = 0.5. Toolkit script: Fig6_MoGGauss_graphicalDemo.m. ***D***, Full psychometric curves for the noise variances used in panels ***A–C***, showing the probability that the observer reports “yes” as a function of the stimulus X_2_. The points labeled *A*, *B*, *C* represent the sum of the probability above the diagonal in panels ***A–C***.

To numerically estimate the ideal observer’s prior from a set of experimental 2AFC data using a mixture of Gaussians prior, we can again use the general form of the log-likelihood defined in Equation 31. Here, $p(“\text{yes}”\text{|}x_{\text{1},t},x_{\text{2},t})$ is defined with the general solution in Equation 25, and the decision rule $T(m_{1},m_{2})$ follows the definition in Equation 21. Since the estimate distributions are not guaranteed to be Gaussian, there is no simple analytical solution like there was in the single Gaussian prior model. Thus, these equations must be evaluated numerically by calculating $p(x_{2}>x_{1}|m_{1},m_{2})$ for each measurement pair on the 2D support to define $T(m_{1},m_{2})$, as illustrated previously in Figure 4. Once the boundary defined by this decision rule is found, we can simply integrate the joint distribution $p(m_{1},m_{2}|x_{1},x_{2})$ above this boundary to determine $p(“\text{yes}”\text{|}x_{\text{1},i},x_{\text{2},i})$ and evaluate the model likelihood. This process is again outlined graphically in Figure 6, with the white line now denoting an example decision boundary for an observer with a mixture of Gaussians priors.

Compared with the single Gaussian case, the mixture of Gaussians decision boundary can be nonlinear for a few reasons. One reason is the dependence of each adjusted weight ${\tilde{w}}_{i}$ on *m*: the weight of the shrinkage factor for each prior component decreases with a greater difference between the component mean and the likelihood mean. As a result, the perceptual bias that the prior exerts is different at different points along the stimulus domain. Nonlinear decision boundaries can also emerge when the prior is bimodal, with measurements biased in different directions depending on which mode is closest. A function for numerically evaluating $p(“\text{yes}”\text{|}x_{\text{1},i},x_{\text{2},i})$ is included with the toolkit (calcMoGPFxn_Numeric.m).

As noted for the point estimation case with a mixture of Gaussians prior, this numerical calculation can be computationally expensive. We can, however, leverage the approximate analytical expression for the estimate distribution to define an approximate expression for the categorical data collected in a 2AFC experiment. The reason this is possible is that with this approximation, the two-point estimate distributions are Gaussian. Using the Bayesian least squares estimate ${\hat{x}}_{BLS}$ defined in Equation 40, we can generalize the decision rule $T(m_{1},m_{2})$ in Equation 23 to an observer with a Mixture of Gaussians prior:

(51) $$T(m_{1},m_{2})\approx \{\begin{array}{ll} 1 & \sum_{j=1}^{C}{\tilde{w}}_{j}(x_{2})(\alpha_{j}m_{2}+{\tilde{\nu }}_{j})>\sum_{i=1}^{C}{\tilde{w}}_{i}(x_{1})(\alpha_{i}m_{1}+{\tilde{\nu }}_{i})\\ 0 & \text{otherwise} \end{array}.$$

Note that we index the modified weights and means differently for the two stimuli (*i* for *x*_1_ and *j* for *x*_2_) since these parameters of the posterior components are defined by both the prior components and the likelihood parameters, which differ whenever *x*_1_ is different from *x*_2_. As before, we can derive an analytical (although approximate) solution to the psychometric function for the mixture of Gaussians approach using Equation 29, with the exception of substituting in the approximate estimate distribution $p({\hat{x}}_{BLS}|x)$ from Equation 49:

(52) $$p(“\text{yes}”\text{|}x_{\text{1}},x_{\text{2}})\approx \Phi \left(\frac{\sum_{j=1}^{C}{\tilde{w}}_{j}(x_{2})(\alpha_{j}x_{2}+{\tilde{\nu }}_{j})-\sum_{i=1}^{C}{\tilde{w}}_{i}(x_{1})(\alpha_{i}x_{1}+{\tilde{\nu }}_{i})}{\sqrt{\sigma_{1}^{2}{\left(\sum_{i=1}^{C}{\tilde{w}}_{i}(x_{1})\alpha_{i}\right)}^{2}+\sigma_{2}^{2}{\left(\sum_{j=1}^{C}{\tilde{w}}_{j}(x_{2})\alpha_{j}\right)}^{2}}}\right).$$

#### Code accessibility

The code is included as Extended Data 1 and is available at https://github.com/tsmanning/bayesIdealObserverMoG.

## Results

In this section, we will demonstrate that there are a number of ways to maintain the flexibility of the mixture of Gaussians approach while reducing the total number of parameters describing the prior, and then show that this approach can be used to fit leptokurtotic and bimodal distributions. Lastly, we show that the approximate 2AFC solution remains close to the numerical solution for a range of model parameters constrained to realistic values. Although we do not go into detail here about how to generate synthetic estimate or 2AFC data using a Bayesian ideal observer framework, we include some example code in the toolkit about how one might benchmark implementations of an observer model with a mixture of Gaussians prior interactiveNumTrialsVSaccuracy.m.

### Prior estimation error using mixture of Gaussians model with point estimation data

Theoretically, a mixture of Gaussians could fit an infinite number of prior shapes given enough Gaussian components in the model. But the number of model parameters increases by three for each additional component, potentially requiring large amounts of data to obtain reliable fits. Further, unrestricted models will likely be nonconvex with multiple local optima. These characteristics extend the number of iterations needed to find the global optimum of the log-likelihood objective functions at best and make it unlikely or impossible to find the global optimum at worst. In practice, unrestricted forms of the mixture of Gaussians model will likely need multiple optimization runs with different starting parameters to reliably minimize the log-likelihood functions. There are a few ways to maintain the flexibility of the mixture of Gaussian approach while reining in the number of parameters in the model.

**Figure 7.**
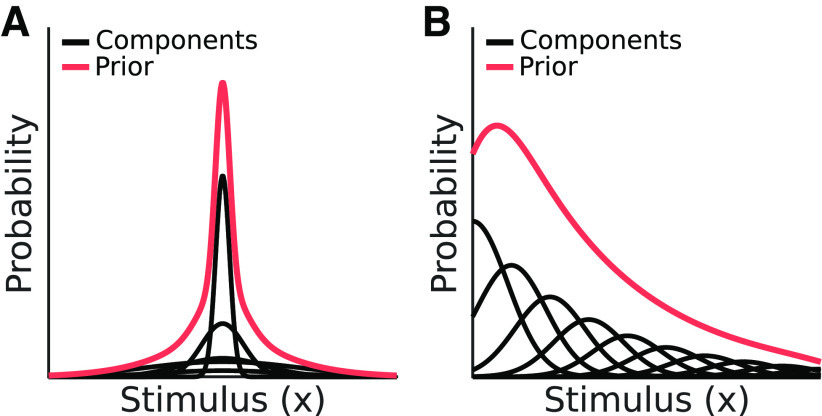
Two example methods for reducing the number of parameters to optimize when inferring an observer’s prior. ***A***, A leptokurtotic prior centered on zero formed by a mixture of zero-mean Gaussian components. ***B***, A skewed prior formed by a mixture of Gaussian components with fixed positions and widths. Toolkit script: Fig7_MoGConstrainedFitting.m.

In sensory subdomains where there is evidence that the probability of some stimulus values monotonically decreases with stimulus magnitude, such as the spectral content of retinal images (Field, 1987; Dong and Atick, 1995), we can reduce the number of parameters by a third in our ideal observer model by fixing all component means at zero. This allows us to model long-tailed distributions as can be seen in Figure 7*A*, and in fact, any distribution that is a member of the exponential power family with a peak at zero and power 1 ≤ *p* ≤ 2 can be approximated with enough components (West, 1987).

If there is not sufficient evidence that the true distribution of stimulus power in the environment is either symmetric or zero-peaked, one can take an alternative approach of tiling the components (Fig. 7*B*). Here, one defines a fixed number of components, their means, and their SDs and fits only the weights of the tiled components to the data. In this way, the mixture of Gaussians can approximate a prior with a peak at an arbitrary location, skewness, and kurtosis. This approach has been used previously with large numbers of components to approximate a “nonparametric” reconstruction of a complicated prior (Acerbi et al., 2014).

Here, we demonstrate proof of principle for both approaches by generating a synthetic dataset of 1000 point estimates using a zero-centered, non-Gaussian prior and a bimodal prior, and then recovering estimates of these priors using the mixture of Gaussians ideal observer model and the constraints illustrated in Figure 7.

**Figure 8.**
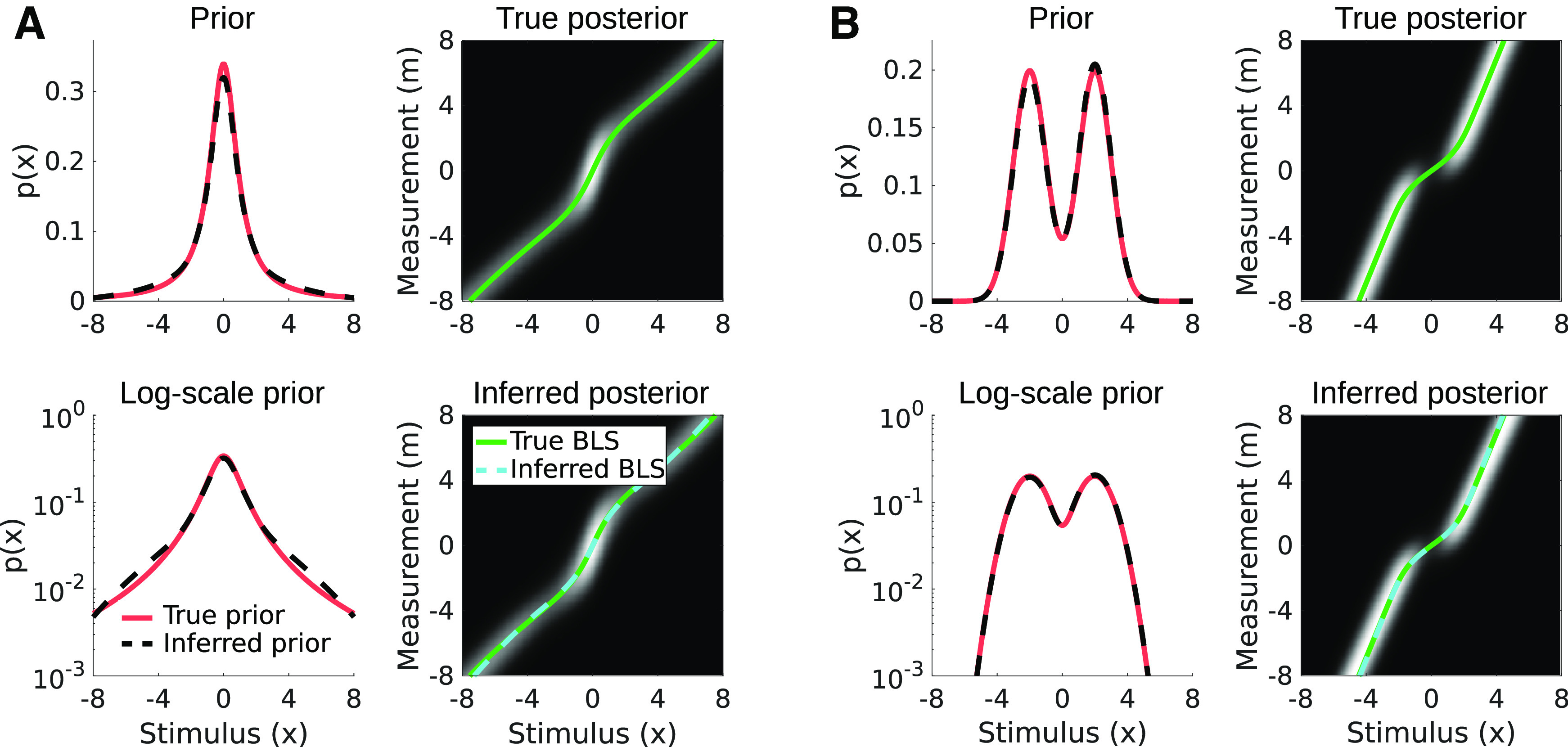
Mixture of Gaussians model fitting to non-Gaussian priors. ***A***, Inferring the shape of a Cauchy prior from a set of 1000 point estimates. Top left, True prior in red and inferred prior in dashed black. Bottom left, The same, but on a semilog axis. Top right, Posteriors for a set of stimuli and measurements, as well x_BLS_ for each posterior (green line). Bottom right, Set of posteriors and x_BLS_ inferred from the data using the mixture of Gaussians model. ***B***, Inferring the shape of a bimodal prior from a set of 1000 point estimates. Conventions are the same as in panel ***A***. A slight gamma correction has been applied to the set of posteriors shown in the 2D plots for visibility. Toolkit scripts: Fig8_MoGtoNonGauss.m and Fig8_MoGtoNonGauss2.m for panels ***A*** and ***B***, respectively.

We first defined a long-tailed prior using a Cauchy distribution $p(x)=1/\pi (1+x^{2})$. We generated individual point estimates by numerically calculating the posteriors for a range of different measurement values as seen at the top right in Figure 8*A* and calculating ${\hat{x}}_{BLS}$ for each measurement. We used this matched set of measurements and Bayes estimates as a look up table, and generated the synthetic dataset of 1000 trials by randomly selecting a stimulus value, adding Gaussian noise to obtain a measurement, and then selecting a matched estimate by interpolating between the previously calculated estimate values. From these values, we estimated the Cauchy prior using a restricted form of the mixture of Gaussians ideal observer model in which we defined six Gaussian components with a set of fixed *γ_i_* on the range $\left[2^{-2},2^{3}\right]$ and all component means *ν_i_* fixed at zero. Thus, the only observer parameters free to vary were the component weights *w_i_*, and the measurement noise level σ which was constant for all simulated stimuli (that is, we are assuming the stimulus properties that may affect this measurement noise are held constant throughout the experiment). The best fitting parameters $\theta =\{w_{i},\sigma \}$ were obtained through numerical optimization by numerically estimating $T^{-1}(\hat{x})$ to obtain a set of {*m_i_*} from the dataset of $\left\{\hat{x}\right\}$ and then minimizing the negative log-likelihood, which is the sum over the individual negative log likelihoods (see Eq. 43). The correspondence between the true prior and the inferred one are shown at the in Figure 8*A*, as well as the correspondence between the true BLS estimates and the ones inferred through MLE. In general, the mixture of Gaussians model closely matches the true prior although each of the basis function components on their own are less kurtotic than their sum.

We then repeated this process using a bimodal prior defined by the normalized sum of two Gaussians $p_{1}(x)=N(\nu_{1}=-2,\gamma_{1}=1)$ and $p_{2}(x)=N(\nu_{2}=2,\gamma_{2}=1)$, and this time using the tiling constraint variation mixture of Gaussians model previewed in Figure 8*B*. Once again, the prior inferred from the data closely corresponds to the true prior, although this correspondence will change depending on the exact spacing and width of the basis functions (Fig. 8*B*).

### Error in mixture of Gaussians analytical approximation with 2AFC data

We will next examine how close the approximate analytical solution is to the numerical solution within a range of observer parameters that matches the biases and sensitivities seen in real human data.

#### Human bias

To get a sense of what a realistic range of biases is in the literature, we consider empircally measured perceptual biases for linear (i.e., noncircular, nonspherical) stimulus domains like speed and distance. For example, in Stone and Thompson (1992), participants performed a 2AFC speed judgment task in which they selected which of two contrast and speed-varying stimuli appeared to move faster. Depending on the contrast ratio between the two stimuli, biases in speed judgments ranged from ∼0.55 to 1.55 times the veridical speed. Similar results were found in later studies that developed Bayesian ideal observer models to explain these biases (Weiss et al., 2002; Stocker and Simoncelli, 2006). An analysis of speed judgments for contrast-varying stimuli in 2D and 3D (Cooper et al., 2016) found a bias of up to ∼1.75 times veridical. In a disparity judgment task, Burge and colleagues reported bias of ∼1.15 (Burge et al., 2010). Thus, we will ensure that the simulated observer parameter models will at least reach these levels in our error analysis. The relationship between bias and observer parameters is straight-forward for a single Gaussian prior and Gaussian likelihood. It is simply the fraction of the shrinkage factors $\alpha_{1}/\alpha_{2}$ for the two stimuli, where the observer is unbiased when the fraction equals one. Referring back to Equation 12, this means we need to select the stimulus likelihood widths $\sigma_{1},\sigma_{2}$ and prior width γ to ensure that the upper and lower bounds of $\frac{\gamma^{2}+\sigma_{1}^{2}}{\gamma^{2}+\sigma_{2}^{2}}$ fall on the range of 0.55–1.75. For mixture of Gaussian priors, the analytical approximation essentially treats the posteriors as Gaussians with SDs defined by $\sigma^{2}{\left(\sum_{i=1}^{C}{\tilde{w}}_{i}(x)\alpha_{i}\right)}^{2}$. This means we can produce human-like biases as long as we select observer parameters such that ${\left(\sum_{i=1}^{C}{\tilde{w}}_{i}(x_{1})\alpha_{i}\right)}^{2}/{\left(\sum_{j=1}^{C}{\tilde{w}}_{j}(x_{2})\alpha_{j}\right)}^{2}$ also falls within this range.

#### Sensitivity

The slope of the psychometric curve at the point of subjective equality (PSE; i.e., the value of *x*_2_ where $p(“\text{yes}”\text{|}x_{\text{1}},x_{\text{2}})=0.5$) is commonly used as a scalar metric to describe observer sensitivity when performing a 2AFC task. The slope has an analytical solution $1/(\sigma_{\text{diff}}\sqrt{2\pi })$ when the psychometric curve is a cumulative normal distribution, which occurs when distribution of differences between estimates $\left\{{\hat{x}}_{1},{\hat{x}}_{2}\right\}$ is a Gaussian $N(\mu_{\text{diff}},\sigma_{\text{diff}})$. This is the case for the single Gaussian prior and the analytical approximate solution for a mixture of Gaussians prior (see Eqs. 29 and 51), but not necessarily for the full, numerically evaluated mixture of Gaussians prior. In psychophysical data, this slope could reasonably range from near-infinite when the task is very easy to zero when the task is impossible to solve and the observer is guessing for all stimulus parameters. Therefore, we will define the range of observer parameters to cover a large range of slopes.

Although there is an infinite range of possible prior configurations to test, we will restrict ourselves here to two useful situations not well fit by a single Gaussian: (1) a prior with only zero-mean components creating a leptokurtotic unimodal distribution and (2) a bimodal prior.

#### Example 1: leptokurtotic unimodal prior

**Figure 9.**
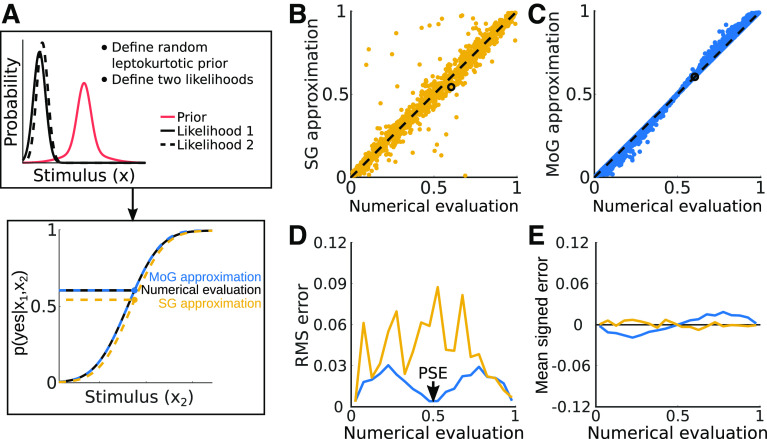
Performance of approximations for fitting heavy-tailed priors. ***A***, Diagram illustrating the pipeline for comparing the mixture of Gaussians (MoG) approximation and a single Gaussian (SG) to a full numerical evaluation of two-alternative forced choice data generated with a MoG prior. ***B***, ***C***, Scatter plots illustrate the relationship between the numerical evaluation of the MoG prior model and the SG and approximate MoG approaches. Black circles indicate the points corresponding to the estimated psychometric function values shown in panel ***A*** for the SG and MoG approximations. ***D***, Square root of the mean squared error (RMS error) for the MoG analytical approximation and the single Gaussian approximation, summarized over 20 bins of the numerical data. ***E***, Mean signed error distributions for both approximations. Note that axis ranges are set to match Figure 10 for comparison. Toolkit script: Fig9_MoGErrorAnalysis.m.

First, we randomly selected a set of stimulus and observer configurations 5000 times (Fig. 9*A*, top). Likelihood means were selected from a uniform distribution ranging from [–1, 1] and SDs $\left\{\sigma_{1},\sigma_{2}\right\}$ were selected from a uniform distribution in the range of 0 < σ ≤ 1. The prior was restricted to two components, both zero-centered, which were both constrained to be broader than the likelihoods. Specifically, *γ_1_* was fixed at $1.1\max(\sigma_{1},\sigma_{2})$ and *γ_2_* was randomly selected from a uniform distribution on the range $\left[1.25\max(\sigma_{1},\sigma_{2}),3.25\max(\sigma_{1},\sigma_{2})\right]$). The weights on these components were chosen randomly from a uniform distribution and normalized such that they summed to one. Fixing *γ_1_* to be only slightly larger than the largest σ ensured that the priors were non-Gaussian and long-tailed, and that the priors produced psychometric functions with a range of biases covering the targeted range (actual biases ranged from 0.55 to 1.83).

Next, we determined the difference between the psychometric curve resulting from the analytical and numerical approaches described in the previous section (Fig. 9*A*, bottom). We compare the approximate solution (Eq. 50) to a numerical evaluation (Eq. 25) for the observer with a mixture of Gaussian prior (blue and black lines). We also compare the best fit single Gaussian to the mixture of Gaussians prior (yellow line). In doing so, we can directly assess the improvement of the approximate mixture of Gaussians approach over the single Gaussian approximation. These results are plotted in Figure 9*B–E*. Figure 9*B*,*C* show the correspondence between the single Gaussian (Fig. 9*B*) and approximate mixture of Gaussians (Fig. 9*C*) models and the numerical evaluations. The points all fall near the identity line, indicated reasonable agreement, but the spread is clearly larger for the single Gaussian model. Figure 9*D* summarizes the errors, showing that the analytical mixture of Gaussians approximation has approximately three times lower RMS error than the single Gaussian fit. The error reduction is most profound about the PSE in the psychometric function, where the analytical and numerical approximations are essentially equivalent. This means that one can precisely estimate observer biases even for non-Gaussian priors in a computationally-efficient manner. The analytical approximation does show a slight tendency to overestimate the upper flank of the psychometric curve and underestimate the lower flank (visible with the mean signed errors; Fig. 9*E*), indicating a bias toward steeper psychometric functions. Thus, when using this approximation to fitting psychometric data of observers with heavy-tailed priors, this will produce prior and/or likelihood estimates that are narrower than the true values.

#### Example 2: bimodal prior

**Figure 10.**
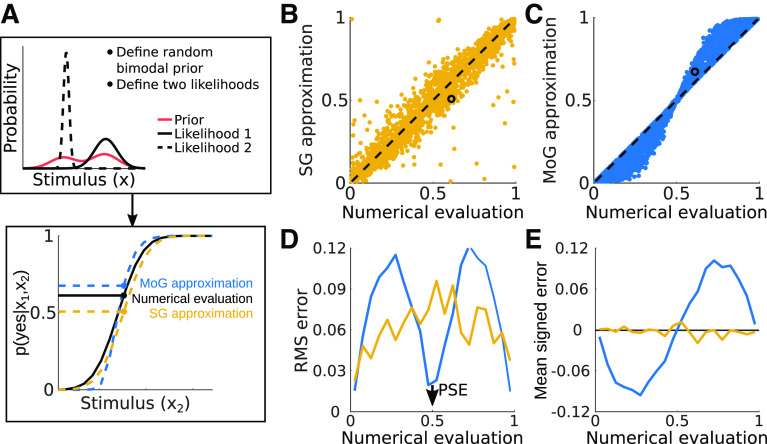
Performance of the analytical approximation in fitting bimodal priors. ***A***, Diagram illustrating the pipeline for comparing the mixture of Gaussians (MoG) approximation and a single Gaussian (SG) before a full numerical evaluation of two-alternative forced choice data generated with a MoG prior. ***B***, ***C***, Scatter plots illustrate the relationship between the numerical evaluation of the MoG prior model and the SG and approximate MoG approaches. Black circles indicate the points corresponding to the estimated psychometric function values shown in panel ***A*** for the SG and MoG approximations. ***D***, Square root of the mean squared error (RMS error) for the MoG analytical approximation and the single Gaussian approximation, summarized over 20 bins of the numerical data. ***E***, Mean signed error distributions for both approximations. Toolkit script: Fig10_MoGErrorAnalysis2.m.

Next, we assess the performance of the mixture of Gaussians analytical approximation for fitting psychometric data from an observer with a bimodal prior. We randomly selected likelihood means and SDs in the same fashion as we did for the zero-mean prior. To define a bimodal, two-component prior on each randomization, we selected the component means $\left\{\nu_{1},\nu_{2}\right\}$ from a uniform distribution where one component was restricted to the range [–1, –0.5] and the other from [0.5, 1]. The component SDs were randomly selected from a uniform distribution in the range $\left[\max(\sigma_{1},\sigma_{2}),1.4\max(\sigma_{1},\sigma_{2})\right]$ to ensure each prior had two distinct peaks. Each component weight was randomly selected and the two were normalized such that they summed to one. As before, we present data from 5000 randomization runs in Figure 10. Overall, the approximate mixture of Gaussians method precisely estimated the location of the PSE (i.e., this method has low RMS error ∼0.5) for the bimodal priors. Compared with the leptokurtotic unimodal case, however, the method shows increases in both RMS error and signed error along other regions of the psychometric function. The end result is that while the analytical approximation can accurately estimate an observer’s bias, it will again tend to overestimate the slopes of the observer’s psychometric functions.

## Discussion

The Bayesian ideal observer framework has proven broadly useful for explaining perceptual phenomena in multiple sensory modalities. For example, a prior that peaks at zero speed (a “slow motion” prior) has successfully predicted systematic biases in judgements of the speed (Weiss et al., 2002; Stocker and Simoncelli, 2006) and direction (Weiss et al., 2002; Sotiropoulos et al., 2011; Rokers et al., 2018) of moving objects. A “light from above” prior about the position of the illuminant in a scene has been used to explain biases in the perceived shape of ambiguously shaded figures (Adams et al., 2004). Similarly, priors for viewing angle, convexity, and alignment between principal lines of curvature and surface contours can explain biases in the interpretation of surface curvature from simple line drawings (Mamassian and Landy, 1998). Other examples of the success of Bayesian perceptual models include prediction of biases in the timing of intervals between discrete events (Sohn and Jazayeri, 2021), the perceived structure in complex moving patterns (Yang et al., 2021), judgments in the orientation of contours (Girshick et al., 2011), and the orientation of surface tilt in natural scenes (Kim and Burge, 2018).

Here, we reviewed the straightforward approach for inferring Bayesian ideal observer models from psychophysical data when it is assumed that priors and sensory noise are Gaussian distributed. Following on a step-by-step formulation of this approach, we then extended the model to include prior distributions described with mixtures of Gaussians. In doing so, we build on previous work that has used mixture of Gaussian priors in other perceptual applications. For example, one group used a mixture of Gaussians to to define the relative probabilities of experimental stimuli and then probed suboptimalities in perceptual inference (Acerbi et al., 2014). Another group used a mixture of Gaussians approach to model human observer priors about homogeneity of orientation to understand biases in visual short-term memory tasks (Orhan and Jacobs, 2014).

Importantly, this mixture of Gaussians extension of the Bayesian ideal observer framework complements and expands on existing approaches for modeling the relationship between natural scene statistics and perceptual priors. First, if perceptual priors indeed match the non-Gaussian distributions of natural stimuli, then using a mixture of Gaussians model of priors may improve how well we predict perceptual biases when stimulus measurements fall on different regions of the stimulus domain, as compared with a single Gaussian model. Second, the mixture of Gaussians approach provides a tool for researchers to constrain Bayesian models using empirically measured stimulus statistics. Bayesian models have faced criticism because of their lack of constraint in how the priors or likelihoods are defined (Jones and Love, 2011; Marcus and Davis, 2013; Rahnev and Denison, 2018). One way to constrain the prior is to assume that the visual system has veridically learned the statistics of natural scenes and these learned statistics are reflected in the prior. In this case, one could define the ideal observer prior with a mixture of Gaussians that matches an empirically measured distribution of scene statistics, forgoing the need to fit the prior from perceptual judgment data. Indeed, several groups have made progress in the estimating the distribution of spectral content in terrestrial scenes (Field, 1987; Dong and Atick, 1995), tilt of objects in natural scenes (Burge et al., 2016), binocular disparity (Sprague et al., 2015), and the spectral content of retinal motion during eye and head movements (DuTell et al., 2020). While the match between estimates of natural statistics and perceptual biases has been investigated previously with numerical methods (Girshick et al., 2011; Sprague et al., 2015), a (relatively) low dimensional parameterization of these stimulus distributions opens up new opportunities for efficiency and experimental investigations.

### Limitations and alternative approaches

Although numerical estimation of the model parameters will find an exact solution with sufficient precision, this is not always possible in practice. Estimating $p(“\text{yes}”\text{|}x_{\text{1}},x_{\text{2}})$ requires summation over many 2D probability mass functions, which must be redefined everytime the ideal observer parameters are changed (e.g.,during numerical optimization). Further, the MLE loss functions for both the numerical and analytical methods defined in this document are likely to be nonconvex and thus potentially prone to falling into a local minimum. This problem can be potentially overcome by initializing the numerical optimization in multiple locations within the loss function hypersurface, although this will add additional computation time to the estimation.

While the approximate analytical method dramatically improves the computational efficiency of the ideal observer parameter estimation, it deviates from the true solution for $p(“\text{yes}”\text{|}x_{\text{1}},x_{\text{2}})$ the further *x*_2_ gets from the point of subjective equality. As shown in Figure 10, this method is also especially prone to errors away from the PSE when the prior or posterior are bimodal. These problems can be mitigated in a few ways. If the approximate analytical method is to be used to adaptively select stimuli during an experiment, the numerical approach can be used after data collection to reach a more accurate solution. If there is good reason a priori to think that an observer’s prior is bimodal (e.g., based on natural stimulus statistics), one can just fall back to the numerical solution.

Throughout this document, we assert that the ideal observer likelihood and measurement distributions are Gaussian along the domain in which the observer encodes the stimuli. Other model parameterizations, however, have been proposed that constrain the likelihood based on physiology and other assumptions, and result in notably asymmetric, non-Gaussian likelihoods (Zhang and Stocker, 2022). We also focus here on stimuli defined along a linear axis (e.g., position, velocity, binocular disparity), and therefore, the methods as presented cannot be directly applied to perceptual judgments about stimuli defined on a circular axis (e.g., orientation, visual motion direction, position of an illuminant). Despite this limitation, previous work has successfully used circular statistics to explain perceptual biases with a Bayesian ideal observer model (Mamassian and Landy, 1998; Burge et al., 2016). As a circular analog of the Gaussian, a mixture of von Mises distributions is a natural extension of the mixture of Gaussians approach.

Finally, we focus here on perceptual priors and not priors involved in decision-making or perception-action contingencies. Decision strategies could presumably affect the loss function as well, if there was an advantage to taking some other summary statistics from the posterior distribution instead of the least squares estimate. The influences of these strategies have been considered elsewhere (Chambers et al., 2019) but are out of the scope of the current work.

In conclusion, many scientific questions about how prior knowledge is incorporated into perceptual judgments and perceptually-guided behaviors remain unanswered. Within the Bayesian framework, for example, do priors vary significantly between observers and do they vary between different tasks? How closely do priors follow from the statistics we can measure empirically from the environment across multiple stimulus domains? How adaptable are priors in response to changing stimulus statistics? A major limiting factor in answering these questions is the accuracy and efficiency with which we can estimate people’s priors from experimental data. Broadening the computational toolkit for experimenters and modelers to address this challenge is an important component of the larger effort to advance our understanding of the transformation from sensation to perception.

10.1523/ENEURO.0144-22.2022.ed1 Extended Data 1 The archive bayesIdealObserverMoG.zip contains MATLAB code for generating the figures in this document, for performing the model comparison presented in Figures 9 and 10, and for fitting psychophysical data from stimulus estimation and 2AFC tasks. Download Extended Data 1, ZIP file.

## Synthesis

Reviewing Editor: Satu Palva, Helsingin yliopisto Decisions are customarily a result of the Reviewing Editor and the peer reviewers coming together and discussing their recommendations until a consensus is reached. When revisions are invited, a fact-based synthesis statement explaining their decision and outlining what is needed to prepare a revision will be listed below. The following reviewer(s) agreed to reveal their identity: Michael Landy.

This is a methods paper that suggests using constrained mixture-of-Gaussian parametric models of priors when using psychophysical data from estimation and 2AFC tasks to learn about observer priors. This is certainly not an unreasonable suggestion, although I’m not as sure about the novelty. The paper is meant somewhat as a how-to and thus spends a bunch of time reviewing material that isn’t new (e.g., the Gaussian case). I found the math to be a bit of a slog, even though this is a bit up my alley ;^) In some spots, I think the math and graphics were confusing and/or incorrect, which doesn’t help.

Details:

103: “note that the prior is constant over m”: This phrase might confuse a reader new to this stuff. Of course, a prior is a function over true values, not over measurements, and you are only saying this because in the figure it is plotted as a function of x and m because you will be multiplying them. Just try not to confuse the innocent ;^)

Eq. 7: \rho is an odd constant, since you are already in a proportional-to situation since you’ve dropped the denominator (p(m)). Indeed, you do use this later in the manuscript, but here it seems pointless, so you might point out that it will be needed later.

Eq. 13: For those that are familiar with the optimal cue-integration typical math, they will be used to thinking of this as a weighted average (\hat{x} = \alpha\m + (1-\alpha)\nu). Your \tilde{\nu} is, in fact, equal to (1-\alpha)\nu, but your equations and explanation never make that clear. The weighted-average idea is easy to understand, so why avoid it?

164-165: “the mean of the measurement distribution no each trial will be equal to m”. That’s not appropriate use of the language. The mean of the measurement distribution on a trial is (for unbiased measurements) equal to the true value x. What you are saying here is a description of the likelihood function p(m|x). A likelihood function, as you no doubt know, is not necessarily also a probability density (i.e., it needn’t integrate to one). As such, although I read it now and then, I would really avoid the term “likelhood distribution", and it’s also not appropriate to talk about its mean, and it definitely is NOT a measurement distribution.

Eq. 14, et seq.: All of this is well known, and yet this is a pretty confusing and obscure development of this well-known Gaussian cue-integration math. Maybe you do it in this fully laid-out notation with changes of variables and such so that you can re-use the notation in the later sections.

Eqs. 22-24: This is unclear. This math is now from the experimenter’s perspective (rather than the observer’s). The model fit involves three parameters: the shape and location of the prior (\gamma, \nu) and the width of the measurement distribution (sensory noise \sigma). First, how you got the minimizing value of \alpha in the running text. It is not possible just to the take the derivative with respect to \alpha, because \alpha depends on \gamma and \sigma, and so does \tilde{\nu}. So, not only is it not possible to derive that expression, but it seems logically circular. Note that in Equation 23, both x and \hat{x} need a subscript i. But again, Eq. 23 is also logically circular, because it defines the ML \hat{\sigma} as a function of \hat{nu}, which in turn is defined based on \sigma. So, this makes no sense to me at all. Of course, maybe I’m confused, but then other readers will be as well.

Eq. 26: This equation only makes sense if you swap the order to dx_2 dx_1 for the integration.

Figure 4: These graphical representations confound two different sets of axes, as best as I can figure out. You label the axes as x_1 and x_2, i.e., true values in the world. Yet, the coloring inside the plots is clearly a measurement distribution for a fixed pair of x’s, so the x- and y-axes are about measurements, not true values (m_1, m_2). As labeled (x_1, x_2), the criterion white line should always be the equality diagonal; the moving white line across panels also only makes sense if the axes are measurements, not true values. Note that some readers may be confused by the plots at the top, because the solid and dashed likelihoods are effectively marginals, and likelihood 2 belongs on the right-hand edge of the plot, not the top. Also, the prior is meant to be Gaussian, but is cut off at zero. Again, avoid saying “likelihood distribution”.

Eq. 31: This looks wrong. You use \nu in the two normals in the first line, but they should be \tilde{\nu} and, in fact, they differ from each other if the two stimuli have different reliabilities, so they don’t cancel in the subtraction.

Eq. 38: the variance should have factor \alpha_i^2

Eqs. 42-43: First, \rho is not “inversely proportional", since the difference from the prior mean is exponentiated, so it’s not proportional to the reciprocal of the difference. Second, you switched from \nu_i to \mu_i going from 42 to 43. There is no \nu_i; the prior doesn’t depend on the trial number. For that matter, there is no need to replace the measurement (m_i) with the mean of the “likelihood distribution", since the latter is an abuse of terminology. The same goes for line 293.

Eq. 47: The 2nd line of Eq. 47 makes no sense. The first line is a correct equality. Should the 2nd line have an “equals” sign up front? Is it equal to the preceding (it doesn’t look like it)?

Eq. 48: Please clarify the notation. First “N” used to be the number of Gaussians in the mixture, but here I think it’s the number of grid points for numerically integrating out the unknown measurement. The last term is the usual change of variables for computing a density, but man, this is an obscure way of presenting this. Please help the reader along, define your notation as you go, clarify the derivation, and don’t reuse notation with multiple meanings. It might even be nice to sum from {i=1} to N.

For the 2AFC case, I don’t think you ever say explicitly that the only way to recover a prior with 2AFC is to do the forced choice between stimuli that differ in reliabiilty (otherwise the prior has absolutely no effect over choices once the measurements arrive).

Figure 6: Has the same wrong axis labels (should be m_1, m_2) and thus the legend should talk about the influence as m varies (not x).

Eqs. 54-56: I didn’t really understand nor check this approximation.

Results: Did the results section ever mention the number of trial per simulation for any of these results? If 20,000 trials is needed to get a good estimate, this procedure is unrealistic. Normally simulation data should give an idea of the reliability of estimates of the prior as a function of the number of trials (and might depend on the specific experimental design as well). If this is meant as an advertisement for the Gaussian-mixture method and the archived code, then one needs to show that the method is useful and feasible. Note, some discussion would be useful of the unconstrained case and its difficulties (all Gaussians free to have whatever means and variances and weights) beyond the explosion in the number of parameters.
